# Flock-dependent exploitation of a limited resource in House Sparrow

**DOI:** 10.1038/s41598-020-64283-y

**Published:** 2020-04-29

**Authors:** Elisa Ligorio, Beniamino Tuliozi, Herbert Hoi, Matteo Griggio

**Affiliations:** 1 Department of Biology, University of Padova, Via Ugo Bassi 58/B, I-35131 Padova, Italy; 2 Konrad Lorenz Institute of Ethology, Department of Integrative Biology and Evolution, University of Veterinary Medicine Vienna, Savoyenstrasse 1a, A-1160 Vienna, Austria

**Keywords:** Evolutionary ecology, Evolution

## Abstract

The performances of different social groups can depend on various characteristics, such as familiarity among their members or the presence of individuals with specific traits. However, it has rarely been investigated how groups perform during an encounter with other conspecifics, even if in the natural environment social groups often run into each other and compete for resources. We investigated whether a certain characteristic of the group (i.e., familiarity) could benefit its members when they are confronted with another group. We designed a novel experimental set-up, creating triads of captive house sparrows *(Passer domesticus)* and examining whether in a situation of competition for limited resources one triad could gain benefits over the other (consume more mealworms, *Tenebrio molitor*). While we did not find an effect of previous familiarity among triad members on the triads’ performances, we discovered a group-based difference in the number of mealworms eaten per capita. Group-mates of the very first individual to eat a mealworm (first feeder) ate more mealworms than those in the opposing triad. First feeder individuals also foraged sooner and more than other birds in a subsequent prey consumption assay. Our results suggest that individual performances were influenced by group membership, even when groups were exploiting the same resource simultaneously.

## Introduction

The variable interactions among individuals living, moving or foraging in a group play a significant role in resource exploitation, disease or information transmission^1–3^. In recent years, increasing attention has been given to the possible differences in performance (i.e. resource use, survival) not only within but also among social groups^4–6^ and their consequent impact on individual fitness^7,8^. How social groups perform can depend on various characteristics, such as the phenotypes of the individuals composing the group^9–12^, the role assumed by particular individuals (*i.e*. keystone individuals^13,14^) or other group properties such as familiarity^15^ or sex-ratio^16,17^. Because of such existing variability among them, groups could enjoy differential benefits according to their characteristics in a particular situation^17,18^, which would translate into benefits for all individuals belonging to that particular group^8,19^. For instance, asocial and bolder individuals can have an advantage during dispersal, as they settle in a novel environment and exploit resources before others, leading to a faster spread of their entire group as well^20^; in other cases, a particular group composition can lead to higher fitness advantages for all its members^6,7^.

An additional factor influencing groups’ performances is familiarity among its members^21–23^: previous experience of groupmates with each other has been shown to give fitness advantages over short^24^ and long^25^ periods of time, particularly in unstable and/or novel environments with scarce resources. Antagonistic interactions are less common among familiar conspecifics^26^; moreover, assessing the threats that unfamiliar individuals might pose can be time-consuming, possibly leading to an increase in individual alert time and stress^27^. In the context of resource acquisition in a novel environment, familiarity has been known to increase the rate of social transmission^28^ and exploratory behaviour and to facilitate social foraging^29,30^.

While there have been studies comparing groups’ performances^31,32^, it has rarely been taken into account how two groups would fare when competing over the same resources together (but see studies on how the process of group’s fusion in fission-fusion societies influences individual social rank^33,34^, associations^35^ and social learning^1^). In the natural environment, however, it is unlikely that groups would not come into contact with each other^36^, or at least share the same resources^37^. While it could happen that groups encountering each other fuse quickly or immediately, thus decreasing the importance of starting out in a specific group, familiarity among group-members could still cause group-linked patterns of movement or foraging^24,38^ strong enough to have an impact on resource acquisition and survival^25^. In this case, not only the performance of one group could be better or worse, but it could also influence the performance of the other group. For example, one group gaining a resource first would mean that individuals of the other group would lose it.

Therefore, our novel experimental set-up attempted to test whether there might be a measurable group-specific advantage in terms of resource acquisition – i.e., a difference in group performance – during a direct confrontation between two groups (but see^39^ for an example of interspecific colony-level confrontation). Moreover, as in the natural environment resources can be a limiting factor, we also implemented a limited resource, so that a benefit gained by one group would create a disadvantage to the other group. We thus created a number of artificial flocks of female house sparrows *(Passer domesticus)*, half entirely composed of already-familiar individuals (familiar flocks) and half entirely composed by unfamiliar individuals (unfamiliar flocks): we then paired them together and examined whether the familiar flocks could gain benefits over the unfamiliar ones in an invasiveness context and on a small-time scale. Afterwards, we tested samples of our population through two different repeated assays, in order to investigate the existence of a relationship between individual performance and measurable behavioural traits. The house sparrow is an opportunistic and sociable passerine, invasive in many areas of the world^40,41^. In winter they reunite in variable-sized flocks that often forage in small sub-flocks, particularly in urban areas. Their social life is thus characterized in this period by repeated fission-fusion dynamics^42^, forcing them to move in small groups and encounter both familiar and unfamiliar individuals, often over clumped and/or limited resources^43^.

We decided to use three sparrows per flock, as a greater number of individuals might have made flock-level phenomena more diluted and difficult to detect. We also decided to use only female individuals, as it has been demonstrated that when tested in a novel environment two female house sparrows familiar with each other differ in key aspect of exploration from a pair of unfamiliar females^22^. Male house sparrows, on the other hand, do not appear to be influenced by familiarity with their companion^22^, a trend that is found in other passerine species^25,44^. Female house sparrows also show a greater tendency to follow other individuals to food sources^45^, and finally, this species disperses during the first year showing a female-biased dispersal pattern^46,47^. As various behavioural traits involved with foraging and exploration have been linked to differences in age, we also decided to test separately adults and juveniles^48^, predicting that, as in other passerine species^49^, younger birds might be faster to explore and acquire food sources, and less neophobic.

In general, we expected to see a flock-based difference in the amount of resource consumed (i.e., an individual would eat more or less depending on its flock). In particular, we set out to test three major hypotheses. Firstly, we hypothesised that familiar flocks would have an advantage over unfamiliar flocks (i.e., they would exploit sooner the food source and consume more of it) because their stronger social connections would facilitate their social exploration. Secondly, we hypothesised that flocks containing the first individuals finding and exploiting the food source might partake in more of the resource. Following others to food sources and novel areas is in fact a paramount behavioural strategy in house sparrows, particularly for females: individuals within a flock strongly differ in their propensity to lead and follow^50^. The presence of a particularly enterprising individual might thus have consequences on the actions of its flock-mates, that if alone would not otherwise venture to certain areas or food sources as quickly^50^. Consequently, if instances of social facilitation were stronger among flock-mates than between individuals from different flocks^24^, there might be an effect of being in the same flock of the first individual to move to the central aviary or of the first individual to acquire the resource (mealworms, *Tenebrio molitor*). Lastly, we hypothesized that roles assumed during the main experiment (such as first to cross into the central aviary or first to forage) might be linked to individual behaviour traits such as greater activity and boldness, e.g. first feeder individuals might be more risk-taking^51^.

## Methods

### Housing and study subject

The study was conducted at the Konrad Lorenz Institute of Ethology (KLIVV, University of Veterinary Medicine) in Vienna, Austria (48 °13′N, 16 °17′). The house sparrows originated from a population kept in mixed-sex outdoor enclosures (mean number of birds/aviary: 10.95 ± 6.80. Measures reported here and henceforward are mean and standard error of the mean), measuring 2 m × 3.9 m and 2.6 m high. We used a total number of 102 female birds. Of these, 42 were born in captivity during the previous breeding season (149 ± 14 days) and had already undergone their post-juvenile moult; the remaining 60 individuals were mature adults (2–3 years old) also born and raised in the same aviaries. Each aviary (from now on “housing aviary”) was equipped with a feeder (consisting of a metal bowl on a wooden pedestal, 1.2 m from the ground), small pine trees, which were used as roosting sites, and branches as additional perching places. All aviaries were provided with food (a mixture of millet, canary seeds, wheat, sunflower seeds, protein-based mash, apple slices and millet sprays hanging from the branches) and water *ad libitum*^52^.

### Experimental design

The trials were conducted in a three-parted outdoor arena, which consisted of three adjoining aviaries linked to each other by two remotely-opened small windows (50×50 cm, 1.4 m from the ground). All birds had previously experienced similar windows in their own housing aviaries and were all able to cross them. The aviaries composing the arena, while identical to the housing aviaries in size and similar in roosting equipment, were however novel to all individuals. The central aviary of the arena was the only one that had a food source, novel to all individuals, consisting of 9 live mealworms placed in three small cups (3 mealworms/cup) above a wooden pedestal. The three aviaries were visually but not acoustically isolated.

All the 102 female individuals were randomly assigned to one of 34 “triads”, i.e. artificially-composed flocks of three individuals of the same age; all triads were tested two at a time, in order to simulate an encounter between two flocks. The two triads facing each other were always of the same age: there were thus 7 trials with opposing triads composed by first-year birds and 10 trials with opposing triads composed by mature individuals. However, triads tested together differed in familiarity: one of the two was composed by individuals that had always (since hatching date) been housed together, hence flock-mates familiar with each other, while the other one was composed by individuals which had never been in contact before the trial (nor visual nor acoustic) hence unfamiliar with each other. While this manipulation of familiarity did not take into account variability in pre-existing social relationships, we reckon that females of this species strongly value the presence of any familiar companions, particularly in potentially stressful situations such as the one they were presented with in this experiment^22^. No bird was tested with siblings, no bird was familiar with any individual of the opposing triad and no individual was tested twice.

**Figure 1:**
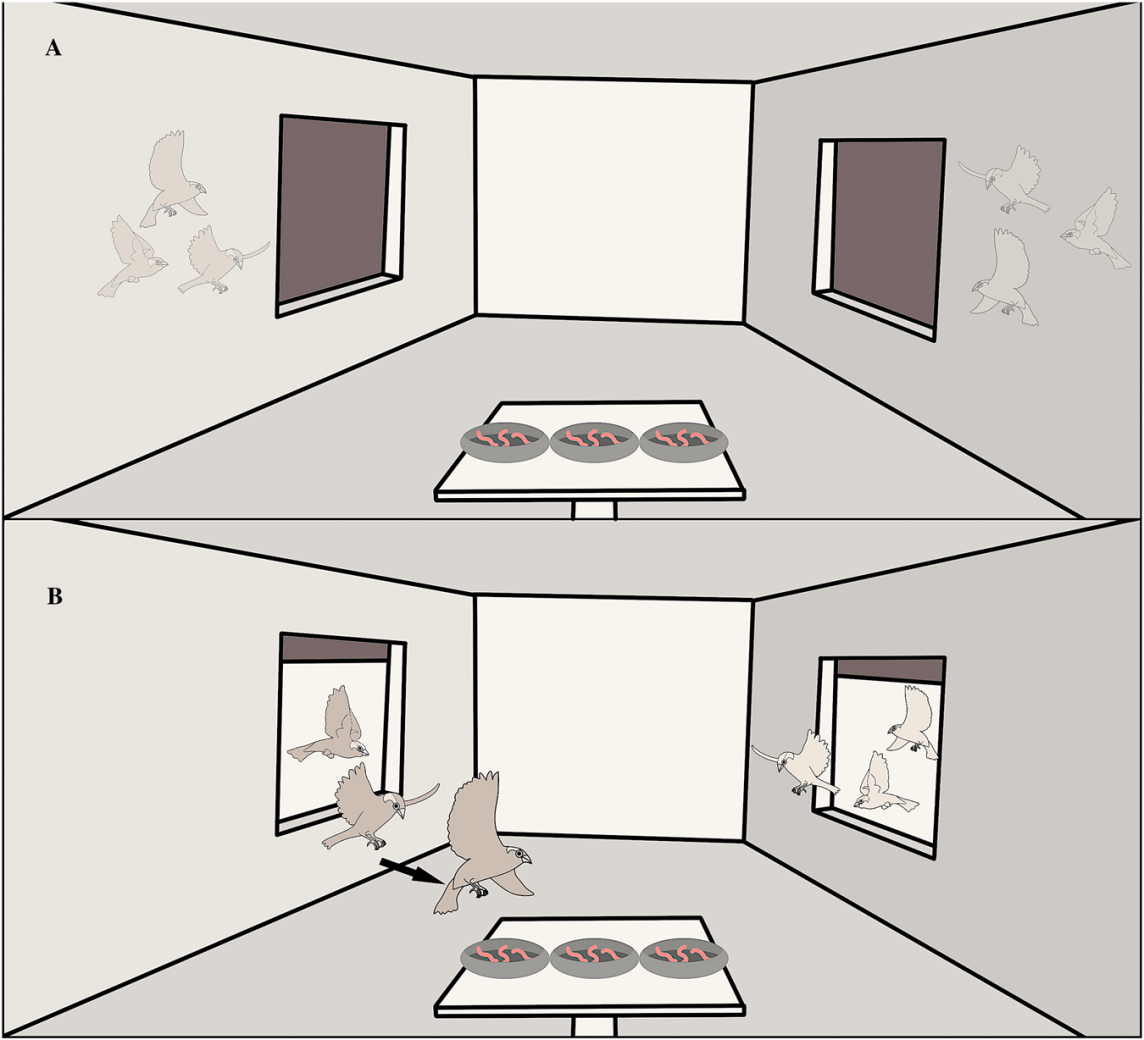
Schematic representation of experimental set-up, allowing two triads of house sparrows to face each other in a central, novel aviary. The novel aviary was equipped with a coveted and limited food source arranged in a novel manner (nine worms in three cups). (**A**) Both triads started in aviaries adjactent to the central aviary, (**B**) Once the windows opened sparrows could freely enter the central aviary. We recorded each individual latency to cross to the central aviary, its latency to feed and the number of mealworms that each individual consumed. The drawing was created by M. Griggio.

The afternoon (1700 hours) before the experiment, the food bowl was removed from the housing aviaries of the individuals scheduled for the trial, in order to standardize the feeding motivation. The trial started the following day at 0800: all study subjects of the two opposing triads were quickly captured with hand-nets and transferred via a small cloth bag to the lateral aviaries, randomly assigning either the left or the right lateral aviary to the familiar triad and the opposite lateral aviary to the unfamiliar triad (Fig. 1A). Here they were given 5 minutes to habituate (the habituation time needed to be short in order to avoid a decrease in the level of unfamiliarity among individuals of newly-formed flocks). After this time, windows opened and the trial started (Fig. 1B). All trials lasted 4 hours as we did not consider a shorter trial to be informative for our purposes^22^. Individuals had in fact to locate and pass through the windows in order to move from the aviary where they had been released to the central aviary. For all individuals we recorded i) their latency to cross the window, ii) their latency to feed, iii) the number of mealworms eaten.

### Individual behaviour assays

In order to measure individual behavioural traits, we tested house sparrows using two different assays: the first one aimed at measuring activity in isolation (‘activity assay’)^53^ while the second one was performed in a group setting, where we tested daily prey consumption rate (‘consumption rate assay’). The first test aimed to provide a measure of activity, while the second aimed to provide a measure of risk-taking behaviour. Activity assays were conducted first, starting a month after the main experiment: as two aviaries had been previously scheduled for different purposes we tested 48 mature birds (80% of all the mature birds tested in the main experiment), and 33 one-year old birds (79% of all the one-year old birds tested in the main experiment). The activity assays were conducted in a cage (Montana-Terenzo, 1 m × 0.5 m and 0.5 m high) equipped with water and seeds on two cups on the front. Each cage was also equipped with two wooden perches, going from the back to the front of the cage. We recorded for all individuals the number of hops within the cage for 10 minutes (from perch to perch or from perch to front), starting 5 minutes after release. Individuals were re-tested after one month in order to measure behavioural repeatability^54^.

The second assay (consumption rate assay) was performed only with one-year old individuals, as we preferred not to disrupt the mature individuals’ social composition any further. The 33 one-year old females were assigned to 11 mixed-sex groups of 6 sparrows each, 3 one-year old males and 3 females. After two months of habituation to their new social groups (habituation started six weeks after the end of the main experiment) we started with the assays. In this assay we measured the amount of resource that each individual would consume in a social setting, just after the introduction of a food source by the experimenter. This measure could be thus interpreted as a proxy of risk-taking behaviour, not unlike the “startle test” widely used in personality research to measure risk-taking behaviour^55–57^, which is based on the latency to go back to a food source after a startle. In our case the startling event was the experimenter entering the aviary and placing the food source inside, which caused birds to fret and fly in the farthest corners; the vicinity of the food source could moreover be considered the riskiest area, as it was where the experimenter had just been. We presented each aviary in the morning (0630–1130; hour of test was randomized across groups every day) with 6 mealworms in a cup. We observed each aviary for 45 minutes, while in that period every other food source was removed from the aviary. We recorded the number of worms that each individual ate and the hour of each feeding event. As the resource was limited, the only individual(s) that could feed were the ones with the shortest latency to approach the food source after the experimenter had left. At the beginning of this assay all birds had already experienced cups with mealworms inside, which could thus not be considered novel food sources: therefore, differences in latency to approach the cups could not be due to differences in experience. The same procedure was repeated for each aviary for 10 days.

### Statistical analysis

#### Main experiment

All data were analyzed with R version 3.2.1^58^. The significance threshold was set at α = 0.05. We used Generalized Linear Mixed Models (GLMMs) to analyze the three dependent variables (individual latency to cross, individual latency to feed, individual number of mealworms eaten). All models were fitted using the ‘glmer’ function within the package lme4 (1.0.5) for R version 3.2.1^59^. Estimates and significance of the fixed effects were obtained using the ‘Anova’ function within the ‘car’ package^60^, while the ‘confint.merMod’ function within the lme4 package was used to obtain 95% confidence intervals via bootstrapping. All non-significant interactions were dropped. Each dependent variable was analyzed using a separate model. As random factors we fitted ‘triad’ nested within ‘trial’ (each trial saw two opposing triads) in all three models. In order to test our hypothesis that individuals belonging to a familiar triad would outperform individuals belonging to an unfamiliar triad we fitted as categorical fixed effects i) age (first-year or third-year) and ii) familiarity (belonging to a triad composed of either familiar or unfamiliar individuals) and their interaction in all three models. To test our second hypothesis, i.e. that individuals would have an advantage if they belonged to the first triad to cross into the central aviary and/or to the first triad to eat a mealworm we had to take into account the effect of social influence on flock-mates behaviour. We thus determined the identity of the very first individual that in every trial crossed the window (‘first crosser’) and the very first individual that ate a mealworm in each trial (‘first feeder’, as in^50^). We created a dummy variable, assigning “1” to each individual in the first feeder and/or first crosser triad and a “0” to every individual in the opposing triad. Thus, we added as independent categorical variables in the models iii) belonging to the triad of the “first crosser” and iv) belonging to the triad of the “first feeder” and their interactions with all other fixed factors. In order to maintain independency of our data we excluded: first feeder individuals from our analysis of the number of mealworms eaten and the latency to eat the first mealworm, and first crosser individuals from the analysis of crossing latency. We analyzed the number of mealworms eaten using poisson distribution (log link), while the latency to cross and the latency eat were modelled with gamma distribution (inverse link). In the models with ‘poisson’ distribution we checked for over-dispersion and whenever it was significant we included an observation-level random effect (OLRE) as detailed in^61^. Birds that did not cross the window or did not eat were assigned a latency of 14400 s.

#### Relation between membership during main experiment and individual behaviour

As the main experiment was performed before the two behavioural assays we decided to test also if being a first feeder or a first crosser in the main experiment had any relation with the expression of individual behavioural traits in the two subsequent behavioural assays (our third hypothesis). We used one dependent variable (number of hops) for the activity assay, and two dependent variables (number of mealworms eaten per day and number of days as first feeder) for the consumption rate assay. Each variable was analyzed using a separate model, focusing on the selection of individuals for which we had measurements of behavioural assays. As both assays were repeated we fitted as random factors ‘identity’ in both models, plus ‘day of test’ (1–10) and ‘aviary’ in the models concerning the consumption rate assay. As categorical fixed effects we fitted i) being or not a first-crosser individual nested within ii) belonging to a first-crosser triad and iii) being or not a first-feeder individual nested within iv) belonging to a first-feeder triad and v) age (only in the model analyzing the hops). We analyzed number of mealworms eaten per day using poisson distribution (log link), level of activity using a Gamma distribution (inverse link) and number of days as a first feeder using binomial distribution (logit link). We also tested for correlation between all dependent variables using the ‘Kendall’ package^62^ applying a false discovery rate correction. To test for repeatability in the individual behavioural traits tested we used package ‘rptR’^63^, which uses parametric bootstrapping to estimate confidence interval and standard errors. We used ‘day’ as fixed effect and ‘group’ as random effect for the repeatability of the consumption rate assay and ‘age’ as the fixed effect for the repeatability of the activity assay.

## Ethical Note

During the course of the study no experimental bird was injured or died. Capture, housing and handling of birds were in accordance with the relevant Austrian laws and were licensed by the government of Vienna (MA 22) license number 114/2012. The experiment reported in this study complies with current laws on animal experimentation in Austria and the European Union. This study was approved by the institutional ethics committee (University of Veterinary Medicine, Vienna) and the national authority according to 8ff (rules) of Law for Animal Experiments Tierversuchsgesetz - TVG, license number GZ 68.205/013-WF/V/3b/2014. The condition and health of experimental birds were monitored on a daily basis.

## Results

### Main experiment

Birds fed in every trial except one, which was thus excluded from the analysis. In the remaining 16 trials 11 birds (11.46%) did not cross during the trial; 42 birds (43.75%) did not eat any mealworm during the trial. In 3 trials the first feeder took the first mealworm when no bird of the other triad had already crossed; however, in only 1 of these 3 trials individuals in the first feeder triad were the only ones to feed. During the 13 other trials when the first feeder took the first mealworm on average 1.362 individuals of its triad and 1.509 individuals of the other triad had already crossed. Birds ate on average 1.438 ± 0.170 mealworms per capita. We found a medium positive correlation between latency to cross and latency to eat the first mealworm (tau = 0.358, p < 0.001), while the number of mealworms eaten was strongly correlated with latency to eat the first mealworm (tau = −0.644, p < 0.001) but only weakly correlated with latency to cross (tau = −0.243, p = 0.001).

Age had no significant effect on the number of mealworms eaten (df = 1, χ^2^ = 1.062, p = 0.303; Table 1) while there was a non-significant trend for mature birds to cross (df = 1, χ^2^ = 2.964, p = 0.085; Table 2) sooner than first-year individual. Previous familiarity with the other triad members did not affect the number of mealworms eaten (df = 1, χ^2^ = 0.794, p = 0.372; Table 1), the latency to eat (df = 1, χ^2^ = 0.023, p = 0.879; Table 3) or the latency to cross (df = 1, χ^2^ = 0.039, p = 0.845; Table 2) of individual birds.

**Table 1:** Effect of ‘age’ (first-year versus mature), ‘familiarity’ (familiar versus unfamiliar), ‘triad of the first crosser’ (first crosser triad versus other triad), ‘triad of the first feeder’ (first feeder triad versus other triad) on the number of mealworms eaten per capita. Coefficients and 95% confidence intervals are presented.

Fixed effect	Comparison	Estimate	2.5% CI	97.5% CI	P value
Intercept		0.268	−0.486	1.190	0.418
Age	First-year versus mature	−0.330	−1.033	0.217	0.303
Familiarity	Familiar versus unfamiliar	0.334	−0.394	1.137	0.373
Triad of the first crosser	First crosser triad versus other triad	−0.286	−1.021	0.651	0.449
Triad of the first feeder	First feeder triad versus other triad	−0.826	−1.629	−0.049	0.011
Random effect		Variance	± SD		
Group		0.001	0.001		
Day		0.001	0.001		
OLRE		0.751	0.767		

P values obtained with Tukey method adjusted for multiple comparisons. ‘Group’, ‘day’ and ‘OLRE’ are fitted as random effects; we show the variance associated with them. The mealworms eaten by first feeders were excluded by the analysis in order to maintain data independence.

**Table 2:** Effect of ‘age (first-year versus mature), ‘familiarity’ (familiar versus unfamiliar), ‘triad of the first crosser’ (first crosser triad versus other triad) on the individual latency to cross into the central chamber.

Fixed effect	Comparison	Estimate	2.5% CI	97.5% CI	P value
Intercept		8.599	7.821	9.382	0.001
Age	First-year versus mature	0.402	−0.590	1.218	0.085
Familiarity	Familiar versus unfamiliar	−0.036	−0.882	0.888	0.844
Triad of the first crosser	First crosser triad versus other triad	−0.073	−0.845	0.784	0.692
Random effect		Variance	± SD		
Group		0.046	0.216		
Day		0.282	0.531		

Coefficients and 95% confidence intervals are presented. P values obtained with Tukey method adjusted for multiple comparisons. ‘Group’ and ‘day’ are fitted as random effects; we show the variance associated with them. The mealworms eaten by first crossers were excluded by the analysis in order to maintain data independence. Results are in the log (not in the response) scale.

**Table 3:** Effect of ‘age’ (first-year versus mature), ‘familiarity’ (familiar versus unfamiliar), ‘triad of the first crosser’ (first crosser triad versus other triad), ‘triad of the first feeder’ (first feeder triad versus other triad) on the individual latency to eat the first mealworm.

Fixed effect	Comparison	Estimate	2.5% CI	97.5% CI	P value
Intercept		9.085	0.302	10.116	0.001
Age	First-year versus mature	0.257	−1.091	1.230	0.149
Familiarity	Familiar versus unfamiliar	−0.025	−1.257	1.066	0.879
Triad of the first crosser	First crosser triad versus other triad	−0.077	−0.738	1.019	0.191
Triad of the first feeder	First feeder triad versus other triad	0.187	−1.629	1.198	0.637
Random effect		Variance	± SD		
Group		0.044	0.210		
Day		0.015	0.121		

Coefficients and 95% confidence intervals are presented. P values obtained with Tukey method adjusted for multiple comparisons. ‘Group’ and ‘day’ are fitted as random effects; we show the variance associated with them. The mealworms eaten by first feeders were excluded by the analysis in order to maintain data independence. Results are in the log (not in the response) scale.

**Figure 2:**
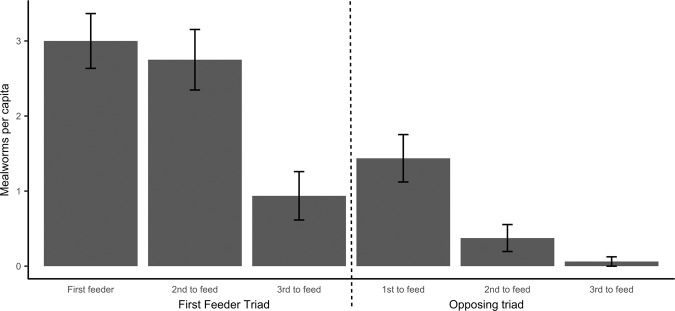
Per capita average number of mealworms consumed during the main experiment. On the left, mealworms consumed on average by individuals of the first feeder triad; on the right, mealworms consumed on average by individual of the opposing triad. Note that first, second and third to feed refer to the ordinal feeding position within the triad. Mean and standard error of the mean are shown.

Out of 144 mealworms available in total 138 were eaten; first feeders ate 48 mealworms (on average 3.000 ± 0.442 mealworms per capita; Fig. 2), individuals belonging to the first feeder’s triad ate 55 mealworms in total (on average 1.719 ± 0.324 mealworms per capita; Fig. 2) and individuals belonging to the other triad ate 35 mealworms in total (on average 0.730 ± 0.174 mealworms per capita; Fig. 2).

**Figure 3:**
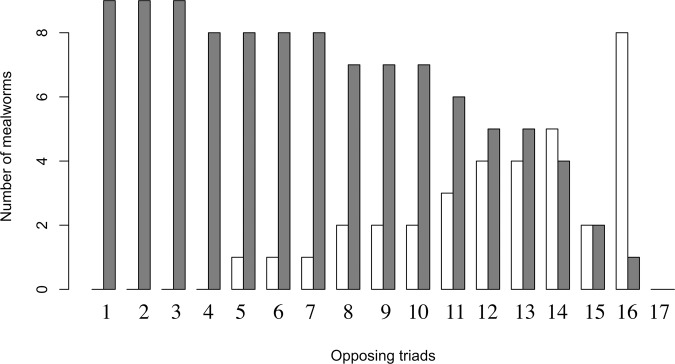
Total number of mealworms acquired by each triad. Bars laying next to each other represent triads tested together (same trial). In dark grey, number of mealworms consumed by first feeder triads. In white, number of mealworms consumed by their opposing triad.

Having the first feeder as a group-mate increased significantly the number of mealworms eaten per capita (df = 1, χ^2^ = 6.480, p = 0.011; Table 1, Fig. 3), but did not affect the latency to take the first mealworm (df = 1, χ^2^ = 1.713, p = 0.191; Table 3). On the other hand, belonging to the first crosser triad did not affect the crossing latency (df = 1, χ^2^ = 0.157, p = 0.692; Table 2) or had any effect on the number of mealworms eaten (df = 1, χ^2^ = 0.572, p = 0.449; Table 1) or on the latency to feed (df = 1, χ^2^ = 0.223, p = 0.637; Table 3).

### Relation between membership during main experiment and individual behaviour

The level of activity each individual fell in was weakly but significantly repeatable (R = 0.196, p = 0.028). Triads did not differ in their composition of behavioural traits, i.e. there was no difference in the number of hops between first-crosser individuals, first-feeder individuals and all the others, nor there was any difference in the number of hops between individuals belonging to first-feeder, first-crosser, familiar or unfamiliar triads (df = 1, all χ^2^ < 0.884, all p > 0.347).

**Figure 4:**
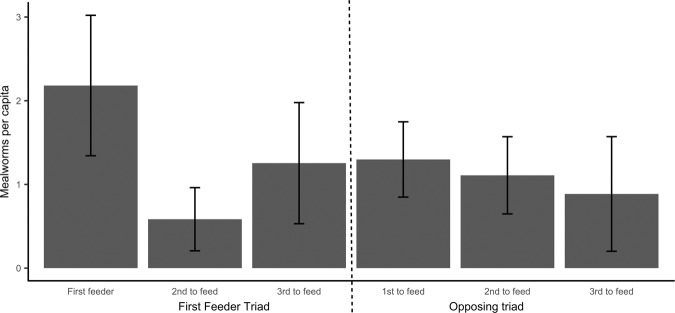
Per capita daily average number of mealworms consumed during the consumption rate assay. On the left, mealworms consumed on average by individuals of the first feeder triad (relative to the main experiment); on the right, mealworms consumed on average by individual of the opposing triad. Note that first, second and third to feed refer to the ordinal feeding position within the triad during the main experiment. Mean and standard error of the mean are shown.

In the consumption rate assay however birds eating more worms were also first feeders more often than their flock-mates (tau = 0.971, p < 0.001): the number of mealworms eaten during the consumption rate assay was repeatable across the 10 days (R = 0.442, p < 0.001). Birds that were first feeders in the main experiment ate more mealworms (df = 1, χ^2^ = 4.705, p = 0.030, Fig. 4) and showed a tendency to be first feeders also in the consumption rate assay (df = 1, χ^2^ = 3.521, p = 0.063) while first-crosser individuals, birds belonging to first-crosser triads or first-feeder triads did not differ on average from their counterparts in the opposing triads in neither variable (df = 1, all χ^2^ < 0.198, all p > 0.239).

## Discussion

To our knowledge, our experiment gives possibly the first evidence of a variable performance between two social groups facing each other in captivity. While we did not find any effect of previous familiarity among triad members on the triad’s performances, we discovered a group-based difference in the number of mealworms eaten per capita: birds belonging to the triad of the first feeder ate significantly more mealworms than those in the opposing triad. As the resource was limited and easily depletable, if a triad consumed more of the resource individuals belonging to the opposite one would have less of it to exploit. We found no difference in the composition of the opposing triads relatively to two individual behavioural traits; however, birds that were first feeders during the main experiment consumed more mealworms and tended to forage first also in the consumption rate assay.

During our trials, first feeders on average acquired also the most mealworms per capita: as individuals virtually never took more than one mealworm at once (Authors’ personal observation), this means that these individuals returned to the feeder more than the others. Nevertheless, the first feeders’ triad-mates also consumed more food items than the individuals in the opposite triad: interestingly, the two triad-mates of the first feeder ate on average more mealworms than all three sparrows in the other triad combined. However, while first feeder birds consistently acquired more worms also in the subsequent repeated consumption rate assay, their triad-mates did not (Fig. 4); they consumed more mealworms than the other sparrows only during the main experiment. This could be due to several non-excluding factors, depending on the dynamic of the social interactions and following movements. It might be argued that a triad entering first in the central chamber could have acquired more of the resource before the opposing triad could even enter. However, this happened only in three out of 17 trials; instead, in the remaining 14 trials when the first feeder took the first mealworm approximately the same number of individuals of both triads had already crossed into the central aviary. Moreover, belonging to the triad of the first crosser did not have any influence on the number of mealworms acquired; crossing into the central aviary appeared to happen either because of greater individual activity or sheer chance. This might be also supported by the fact that first crossers did not differ on average from the other sparrows in neither subsequent assay.

Acquiring mealworms on the other hand was possibly a more purposeful activity, linked to decreased neophobia^64^. Thus, the difference in mealworms per capita shown in the main experiment might be attributed to the triad-mates of the first feeder following it to the food source more readily than the individuals of the other triad. In other words, birds that were already present in the central room and potentially also able to acquire mealworms after the first feeder apparently did it to a lesser extent when the first feeder belonged to the opposite triad. This might mean that, with respect to following behaviour, there was a difference between how individuals regarded their triad-mates and those in the opposing triad.

As the average crossing latency was quite long, individuals may have interacted with each other during that time, thus developing a familiarity with their triad-mates^65^ sooner than expected. This possibly canceled out the effects of previous unfamiliarity with their triad-mates, which in fact did not influence our results: in Tuliozi *et al*. 2018^22^ pairs of unfamiliar house sparrows habituated to each other within the second hour of interaction. On the other hand, the opposing triads were composed of individuals completely unfamiliar to each other when both groups entered the central aviary. This would explain why only triad-mates of the first feeder ate more mealworms: they possibly reacted more strongly to the cue of their triad-mate landing on the pedestal and approaching the food source, as it was an individual that they had – even if relatively briefly – already developed a relationship with and started following. This is consistent with what is known from other studies of this species: social facilitation and following behaviour in house sparrows are vital activities and familiarity among individuals moving together does play a significant role during exploration^22^. Several studies have shown that in other species the decision to move and to join group-mates is conditioned by the network of social relationships and the decision of close partners^66,67^. In particular, a study on three-spined stickleback found that individuals tended to discover a food patch sooner if a familiar individual from their group had previously done so^24^. Moreover, in a previous experiment with an unlimited hidden food source the individuals closely associated with the first feeder gained access to the food source before the others^50^. In our experiment, resources were limited and in fact association with the first feeder led to a difference in the quantity of resource consumed, i.e. to a definite benefit. Consequently, the opposing triad found itself at disadvantage: a greater number of mealworms consumed by one triad meant a lower number of mealworms consumed by the other. We also cannot exclude the possibility of a monopolization of the feeding cups by the first-feeder triad^68^: while aggressive interactions were rarely observed (Authors’ personal observations), the presence of an individual of another triad on the feeder might have been a deterrent for the opposite triad to start foraging.

The variability in individual phenotype (i.e. personality traits such as boldness, exploratory behaviour) is deeply linked to the individual latency to feed in a social context^6,69^. Bolder individuals are often shown to display greater moving initiative, whereas shyer individuals tend to follow conspecifics more^70,71^. Nevertheless, we could not investigate if there was an influence of individual behavioural traits on the sparrows performances in the main experiment and we did not find any evidence for a difference in activity between first feeders and other birds when we tested them in the activity assay. However, during the consumption rate assay one-year old birds that were first feeders in the main experiment again consumed more mealworms and foraged sooner. As the consumption rate assay was conducted after the main experiment it is possible that individual experience might have influenced the acquisition of resources; individuals that foraged successfully in the main experiment could have been more eager to take advantage of the food source. However, the consumption rate assay was performed three months after the main experiment (which lasted only one morning) and in the meantime all birds had had access to cups with mealworms inside: for this reason we reckon that the variability was not due to differences in experience with the cup but rather to a specific behavioural trait.

First feeders individuals were thus faster to acquire the food source in both social contexts, suggesting a consistency in their role within the two very diffferent groups. Approaching and exploiting a food source after the experimenter had tampered with it can be considered a proxy for risk-taking behaviour (the sooner an individual approaches a potentially “risky” food source, the more risk-taker it is)^55^, a trait often linked with exploratory behaviour^56^. However, since we did not manipulate the triads’ composition, we could not further investigate the influence of this behavioural trait on their performance during the main experiment. The triads’ performances might in fact not be a function of the number of bold individuals within them, but the complex result of various different factors; highly performing groups for example might be composed of a mix of different traits^6^. We can however hypothesize that one factor explaining the difference between the triads during the main experiment might have been the personality of only some of the individuals within them^13^. As having precedence to eat gave an advantage in both scenarios, following closely a bold group-mate might have considerably sped up the feeding process^10^. We could thus also speculate that in this species differences among groups might be linked to differences in the phenotype of their boldest individuals^7,8^. In fact, while we detected a posteriori that first feeder birds were indeed either bolder or less risk-averse^57,68^, their entire triads were not, on average, composed of more risk-taker individuals than the opposite ones.

In conclusion, our results appear to suggest a foraging pattern based on both individual characteristics and flock membership^69,72^. Bolder individuals might have led their entire group to acquire a resource sooner^19^, but boldness alone did not influence resource acquisition; moreover, we showed that differences at flock level can lead to variable individual benefits when two flocks are briefly together and competing for the same limited resource, even in fission-fusion societies such as the house sparrows’. We tested this only on a short time-scale, that is, however, the time scale at which many feeding events on limited resources happen. It must also be taken into account that flock size in this species is highly variable: the effect that we observed might be linked to the low number of individuals (three) in each group, while in larger groups dynamics might differ^73^.

These results indicate the necessity to focus not only on individual characteristics and traits when considering the processes of novel environment exploration^74^, dispersal^75,76^ or invasion^77^ in a sociable species, but also on how these characteristics and strategies interact in a context of multiple flocks. In the future it might be worthwhile to investigate how different phenotypes within the group can change the outcome of similar experiments, to test if manipulation of group composition (different mixes of individual characteristics within a group) could determine or influence the performance of an entire group during a merging scenario.

## Data Availability

https://figshare.com/s/821920b86773adcc65dc.
